# Supplementary material for: Suboptimal Performance of Hepatocellular Carcinoma Prediction Models in Patients with Hepatitis B Virus-Related Cirrhosis
Source: Diagnostics (Basel). 2022 Dec 20;13(1):3. doi: 10.3390/diagnostics13010003 (PMC9818663; doi:10.3390/diagnostics13010003)
Supplement: Supplementary file 1 [file diagnostics-13-00003-s001.zip › diagnostics-2017451-supplementary.pdf]

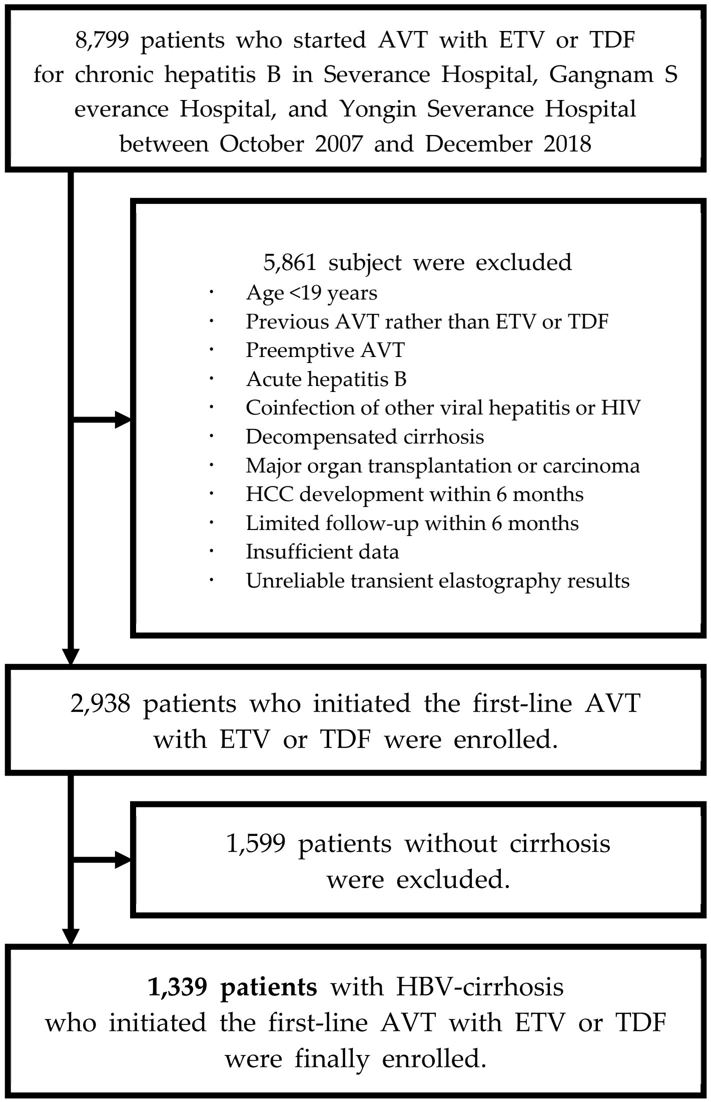
8,799 patients who started AVT with ETV or TDF
for chronic hepatitis B in Severance Hospital, Gangnam S
everance Hospital, and Yongin Severance Hospital
between October 2007 and December 2018
5,861 subject were excluded
· Age <19 years
· Previous AVT rather than ETV or TDF
· Preemptive AVT
· Acute hepatitis B
· Coinfection of other viral hepatitis or HIV
· Decompensated cirrhosis
· Major organ transplantation or carcinoma
· HCC development within 6 months
· Limited follow-up within 6 months
· Insufficient data
· Unreliable transient elastography results
2,938 patients who initiated the first-line AVT
with ETV or TDF were enrolled.
1,599 patients without cirrhosis
were excluded.
1,339 patients with HBV-cirrhosis
who initiated the first-line AVT with ETV or TDF
were finally enrolled.

Supplementary Figure S1. Flowchart of the patients' selection. Abbreviation: AVT, antiviral therapy; ETV, entecavir; TDF, tenofovir disoproxil fumarate; HIV, human immunodeficiency virus; HCC, hepatocellular carcinoma; HBV, hepatitis B virus.

Table S1. Summary of HCC prediction models

**Prediction Model**	**Components**	Risk Stratification (Cumulative Incidence of HCC)
PAGE-B [1]	Age (16–29: 0, 30–39: 2, 40–49: 4, 50–59: 6, 60–69: 8, ≥70: 10)<br>+ Gender (male: 6, female: 0)<br>+ Platelets (10^3/mm^3) (≥200: 0, 100–200: 6, <100: 9)	Low: ≤9 (0%/5Y)<br>Intermediate: 10–17 (3–4%/5Y)<br>High: ≥18 (16–17%/5Y)
Modified PAGE-B [2]	Age (<30: 0, 30–39: 3, 40–49: 5, 50–59: 7, 60–69: 9, ≥70: 11)<br>+ Gender (male: 2, female: 0)<br>+ Platelets (10^3/mm^3) (>250: 0, 200–250: 2, 150–200: 3, 100–150: 4, <100: 5)<br>+ Albumin (g/dL) (≥4.0: 0, 3.5–4.0: 1, 3.0–3.5: 2, <3.0: 3)	Low: ≤8 (0.7%/5Y)<br>Intermediate: 9–12 (5.1%/5Y)<br>High: ≥13 (18.4%/5Y)
Modified REACH-B [3]	Age (1 points for every 5 years from 35 to 65 years of age [0–6 pointes)<br>+ Sex (male: 2, female: 0)<br>+ ALT (IU/L) (15–45: 1, ≥45: 2)<br>+ HBeAg (positivie: 2, negative: 0)<br>+ LS_{TE} (kPa) (<8.0: 0, 8.0–13.0: 2, >13.0: 4)	Low: 0–6<br>Intermediate: 7–11<br>High: 12–13<br>(Incidence was not provided)
CAMD [4]	Age (<40: 0, 40–59: 5, 50–59: 8, ≥60: 10)<br>+ Gender (male: 2, female: 0)<br>+ Diabetes mellitus (diabetic: 1, not diabetic: 0)<br>+ Cirrhosis (presence with age <40: 10, presence with age ≥40: 6, absence: 0)	Low: <8 (0.09–0.27%/3Y)<br>Intermediate: 8–13 (0.85-2.40%/3Y)<br>High: >13 (4.06–10.75%/3Y)
Age–Male–ALBI–Platelets score (aMAP) [5]	$\{(0.06 \times \text{age}) + (0.89 \times \text{sex [Male: 1; Female: 0]})$<br>$+ 0.48 \times ([0.66 \times \log_{10} \text{bilirubin (micromol/L)}]$<br>$+ [-0.085 \times \text{albumin (g/L)}]) - 0.01 \times \text{platelets } (10^3/mm^3) + 7.4 \} / 14.77 \times 100$	Low: 1–50 (0.8%/5Y)<br>Intermediate: 50–60 (4.2%/5Y)<br>High: 60–100 (19.9%/5Y)
Toronto HCC Risk Index (THRI) [6]	Age (<45: 0, 45–60: 50, >60: 100)<br>+ Etiology (Autoimmune: 0, HCV on SVR: 0, steatohepatitis: 54, HBV or HCV: 97, other: 36)	Low: 0–120 (0.3%/Y)<br>Intermediate: 120–240

	+ Gender (male: 80, female: 0)<br>+ Platelets (10^3/mm^3) (>200: 0, 140–200: 20, 80–139: 70, <80: 89)	(1.0%/Y)<br>High: 240–366 (3.2%/Y)
APA-B [7]	Age (<40: 0, 40–59: 1, 50–59: 2, 60–69: 3, ≥70: 4)<br>+ Platelets (10^3/mm^3) (≥130: 0, 100–129: 3, <100: 6)<br>+ AFP (ng/mL) (<5: 0, 5–9: 2, >9: 5)<br>(all variables at 12 months after ETV administration)	Low: 0–5<br>Intermediate: 6–9<br>High: 10–15<br>(Incidence was not provided)
Age, Albumin, Sex, Liver cirrhosis-HCC score (AASL-HCC) [8]	Age (<30: 0, 30–39: 2, 40–49: 4, 50–59: 6, 60–69: 8, ≥70: 10)<br>+ Sex (male: 3, female: 0)<br>+ Cirrhosis (presence: 11, absence: 0)<br>+ Albumin (g/dL) (≥3.5: 0, 2.8–3.4: 3, <2.8: 5)	Low: 0–5 (0.0%/5Y)<br>Intermediate: 6–19 (3.7%/5Y)<br>High: 20–29 (17.6%/5Y)
HCC-Risk Estimating Score in CHB patients Under Entecavir (HCC-RESCUE) [9]	Age (year)<br>+ Gender (male: 15, female: 0)<br>+ Cirrhosis (presence: 23, absence: 0)	Low: ≤64 (0.5%/5Y)<br>Intermediate: 65–84 (14.4%/5Y)<br>High: ≥85 (37.1%/5Y)
Prediction of Liver cancer using Artificial intelligence-driven model for Network - hepatitis B (PLAN-B) [10]	Calculation using the published Python code available in online (https://github.com/vitaldb/hbvhcc/blob/main/predict.ipynb)<br><br>Components: Baseline age, sex, HBeAg positivity, cirrhosis, TDF vs. ETV, HBV DNA titer, ALT, albumin, bilirubin, platelet counts, and races [Asian vs. Caucasian])	Minimal: 0.000–0.075 (0.5%/8Y)<br>Low: 0.075–0.250 (3.8%/8Y)<br>Intermediate: 0.250–0.500 (16.8%/8Y)<br>High: 0.500–1.000 (35.2%/8Y)
CAGE-B and SAGE-B [11]	Based on year 5 variables after AVT<br>Age (23–29: 0, 30–39: 2, 40–49: 4, 50-59: 6, 60–69: 8, ≥70: 10) +<br>① CAGE-B: (LS_{TE} <12 kPa with CHB: 0, <12 kPa with cirrhosis: 3, ≥12 kPa with cirrhosis: 6)<br>② SAGE-B: (LSTE <12 kPa: 0, ≥12 kPa: 5)	CAGE-B<br>Low: 0–5 (0%/5–12Y)<br>Intermediate: 6–10 (1.8%/5–12Y)<br>High: 11–16 (15.4%/5–12/Y)<br><br>SAGE-B<br>Low: 0–5 (0%/5–12Y)<br>Intermediate: 6–10 (4.0%/5–

	12Y)
	High: 11–16 (13.8%/5–12Y)

Abbreviation: HCC, hepatocellular carcinoma; ALT, alanine aminotransferase; HBeAg, hepatitis B e antigen; LS_{TE}, liver stiffness value measured by transient elastography; HCV, hepatitis C virus; SVR, sustained virologic response; HBV, hepatitis B virus; AFP, alpha-fetoprotein; ETV, enteacvir; TDF, tenofovir disoproxyl fumarate

Table S2. Univariate Cox regression analysis for the development of hepatocellular carcinoma.

Variable	Univariate Analysis	
	P Value	Hazard Ratio (95% CI)
Age (year)	<0.001	1.027 (1.012, 1.042)
Male sex	0.837	1.029 (0.785, 1.350)
Diabetes mellitus	0.032	1.464 (1.033, 2.075)
HBeAg positivity	0.012	1.416 (1.079, 1.857)
TDF use (vs. ETV)	0.183	1.209 (0.914, 1.599)
Platelet count ($\times 10^3/\mu L$)	<0.001	0.994 (0.991, 0.996)
Total bilirubin (mg/dL)	0.048	1.051 (1.000, 1.104)
Serum albumin (g/dL)	<0.001	0.469 (0.386, 0.569)
Prothrombin time (INR)	0.014	1.538 (1.092, 2.167)
Aspartate aminotransferase (IU/L)	0.068	1.001 (1.000, 1.001)
Alanine aminotransferase (IU/L)	0.681	1.000 (0.999, 1.001)
Alpha-fetoprotein (ng/mL) (n = 910)	0.004	1.003 (1.001, 1.005)
Liver stiffness value† (kPa)	<0.001	1.025 (1.017, 1.034)
Log HBV DNA (IU/mL)	0.343	1.056 (0.944, 1.180)

†Measured using transient elastography (FibroScan®, EchoSens, Paris, France)

Abbreviation: CI, confidence interval; HBeAg, hepatitis B e-antigen; TDF, tenofovir disoproxyl fumarate; INR, international normalized ratio

Table S3. Comparison of predictive performance between the modified REACH-B and other HCC risk-prediction models

**Scoring Systems**	Differences of Integrated AUC* (95% CI) vs. modified REACH-B
PAGE-B	−0.070 (−0.106, −0.036)
Modified PAGE-B	−0.032 (−0.064, −0.003)
CAMD	−0.090 (−0.123, −0.054)
aMAP	−0.034 (−0.067, −0.001)
HCC-RESCUE	−0.084 (−0.115, −0.052)
AASL-HCC	−0.053 (−0.086, −0.023)
Toronto HCC Risk Index	−0.071 (−0.104, −0.036)
PLAN-B	−0.030 (−0.066, 0.006)
APA-B (*n* = 910) †	-0.011 (-0.055, 0.034) (vs. 0.666 [0.628, 0.703]) †
CAGE-B (*n* = 808) ‡	-0.034 (-0.076, 0.007) (vs. 0.656 [0.616, 0.699]) ‡
SAGE-B (*n* = 808) ‡	-0.020 (-0.063, 0.022) (vs. 0.656 [0.616, 0.699]) ‡

* Integrated AUC were calculated up to 8 years after initiating AVT using 1000 time bootstrap sampling

† APA-B and modified REACH-B were calculated for HCC development after 6 months in 910 patients with baseline alpha-fetoprotein result.

‡ CAGE-B and SAGE-B were calculated for HCC development after 18 months in 808 patients with follow-up transient elastography results after 12 months.

Abbreviation: HCC, hepatocellular carcinoma; CI, confidence interval; AUC, area under the receiver operating characteristic curve

Table S4. Baseline clinical characteristics of the study population who underwent transient elastography after 1 year of antiviral therapy and did not develop HCC within 18 months after antiviral therapy

**Variables**	**Total (*n* = 808)**	**Without HCC (*n* = 694)**	**HCC (*n* = 114)**	*P* Value
Age (year)	54 (47, 59)	53 (46, 59)	56 (49, 61)	0.006
<40	53 (6.6)	50 (7.2)	3 (2.6)	
40–50	217 (26.9)	191 (27.5)	26 (22.8)	
50–60	336 (41.6)	285 (41.1)	51 (44.7)	0.182
60–70	168 (20.8)	138 (19.9)	30 (26.3)	
≥70	24 (3.0)	20 (2.9)	4 (3.5)	
Male sex	447 (55.3)	385 (55.5)	62 (54.4)	0.908
Diabetes mellitus	107 (13.2)	89 (12.8)	18 (15.8)	0.474
HBeAg positivity	314 (38.9)	259 (37.3)	55 (48.2)	0.034
TDF use (vs. ETV)	413 (51.1)	357 (51.4)	56 (49.1)	0.721
Laboratory test results				
Platelet count (×10³/μL)	135 (100, 171)	137 (103, 173)	122 (93, 156)	0.002
Total bilirubin (mg/dL)	0.9 (0.7, 1.3)	0.9 (0.7, 1.2)	0.9 (0.7, 1.4)	0.346
Serum albumin (g/dL)	4.2 (3.9, 4.4)	4.2 (3.9, 4.4)	4.0 (3.6, 4.3)	<0.001
Prothrombin time (INR)	1.02 (0.99, 1.10)	1.01 (0.99, 1.09)	1.04 (0.99, 1.14)	0.022
Aspartate aminotransferase (IU/L)	39 (28, 59)	37 (27, 56)	50 (40, 79)	<0.001
Alanine aminotransferase (IU/L)	38 (25, 62)	37 (25, 59)	48 (33, 82)	<0.001
Alpha-fetoprotein (ng/mL) (*n* = 603)	4.49 (2.73, 8.35)	4.02 (2.59, 7.39)	6.44 (4.56, 19.1)	<0.001
Liver stiffness value† (kPa)	10.8 (7.4, 16.7)	10.3 (7.1, 15.9)	12.9 (9.3, 18.4)	<0.001
1 year after AVT (kPa) (*n* = 808)	8.8 (6.3, 13.1)	8.6 (6.1, 12.4)	11.6 (8.4, 16.1)	<0.001
Follow-up and treatment duration (month)	59.2 (39.5, 76.7)	62.0 (41.0, 77.1)	48.1 (29.9, 69.4)	<0.001

Values are expressed as a n (%) or median (interquartile range).

†Measured using transient elastography (FibroScan®, EchoSens, Paris, France)

Abbreviations: HCC, hepatocellular carcinoma; TDF, tenofovir disoproxil fumarate; ETV, entecavir; HBeAg, hepatitis B e antigen; AST, aspartate aminotransferase; ALT, alanine aminotransferase; INR, international normalized ratio

References

1. Papatheodoridis, G.; Dalekos, G.; Sypsa, V.; Yurdaydin, C.; Buti, M.; Goulis, J.; Calleja, J.L.; Chi, H.; Manolakopoulos, S.; Mangia, G. *et al.* Page-b predicts the risk of developing hepatocellular carcinoma in caucasians with chronic hepatitis b on 5-year antiviral therapy. *Journal of hepatology* **2016**, *64*, 800-806.
2. Kim, J.H.; Kim, Y.D.; Lee, M.; Jun, B.G.; Kim, T.S.; Suk, K.T.; Kang, S.H.; Kim, M.Y.; Cheon, G.J.; Kim, D.J. *et al.* Modified page-b score predicts the risk of hepatocellular carcinoma in asians with chronic hepatitis b on antiviral therapy. *Journal of hepatology* **2018**, *69*, 1066-1073.
3. Jung, K.S.; Kim, S.U.; Song, K.; Park, J.Y.; Kim, D.Y.; Ahn, S.H.; Kim, B.K.; Han, K.H. Validation of hepatitis b virus-related hepatocellular carcinoma prediction models in the era of antiviral therapy. *Hepatology (Baltimore, Md.)* **2015**, *62*, 1757-1766.
4. Hsu, Y.C.; Yip, T.C.; Ho, H.J.; Wong, V.W.; Huang, Y.T.; El-Serag, H.B.; Lee, T.Y.; Wu, M.S.; Lin, J.T.; Wong, G.L. *et al.* Development of a scoring system to predict hepatocellular carcinoma in asians on antivirals for chronic hepatitis b. *Journal of hepatology* **2018**, *69*, 278-285.
5. Fan, R.; Papatheodoridis, G.; Sun, J.; Innes, H.; Toyoda, H.; Xie, Q.; Mo, S.; Sypsa, V.; Guha, I.N.; Kumada, T. *et al.* Amap risk score predicts hepatocellular carcinoma development in patients with chronic hepatitis. *Journal of hepatology* **2020**, *73*, 1368-1378.
6. Sharma, S.A.; Kowgier, M.; Hansen, B.E.; Brouwer, W.P.; Maan, R.; Wong, D.; Shah, H.; Khalili, K.; Yim, C.; Heathcote, E.J. *et al.* Toronto hcc risk index: A validated scoring system to predict 10-year risk of hcc in patients with cirrhosis. *Journal of hepatology* **2017**,doi:10.1016/j.jhep.2017.07.033.
7. Chen, C.H.; Lee, C.M.; Lai, H.C.; Hu, T.H.; Su, W.P.; Lu, S.N.; Lin, C.H.; Hung, C.H.; Wang, J.H.; Lee, M.H. *et al.* Prediction model of hepatocellular carcinoma risk in asian patients with chronic hepatitis b treated with entecavir. *Oncotarget* **2017**, *8*, 92431-92441.
8. Yu, J.H.; Suh, Y.J.; Jin, Y.J.; Heo, N.Y.; Jang, J.W.; You, C.R.; An, H.Y.; Lee, J.W. Prediction model for hepatocellular carcinoma risk in treatment-naive chronic hepatitis b patients receiving entecavir/tenofovir. *Eur J Gastroenterol Hepatol* **2019**, *31*, 865-872.
9. Sohn, W.; Cho, J.Y.; Kim, J.H.; Lee, J.I.; Kim, H.J.; Woo, M.A.; Jung, S.H.; Paik, Y.H. Risk score model for the development of hepatocellular carcinoma in treatment-naïve patients receiving oral antiviral treatment for chronic hepatitis b. *Clinical and molecular hepatology* **2017**, *23*, 170-178.
10. Kim, H.Y.; Lampertico, P.; Nam, J.Y.; Lee, H.C.; Kim, S.U.; Sinn, D.H.; Seo, Y.S.; Lee, H.A.; Park, S.Y.; Lim, Y.S. *et al.* An artificial intelligence model to predict hepatocellular carcinoma risk in korean and caucasian patients with chronic hepatitis b. *Journal of hepatology* **2022**, *76*, 311-318.
11. Papatheodoridis, G.V.; Sypsa, V.; Dalekos, G.N.; Yurdaydin, C.; Van Boemmel, F.; Buti, M.; Calleja, J.L.; Chi, H.; Goulis, J.; Manolakopoulos, S. *et al.* Hepatocellular carcinoma prediction beyond year 5 of oral therapy in a large cohort of caucasian patients with chronic hepatitis b. *Journal of hepatology* **2020**, *72*, 1088-1096.
